# Oxidation of Hydrogen Sulfide by Quinones: How Polyphenols Initiate Their Cytoprotective Effects

**DOI:** 10.3390/ijms22020961

**Published:** 2021-01-19

**Authors:** Kenneth R. Olson, Yan Gao, Karl D. Straub

**Affiliations:** 1 Indiana University School of Medicine—South Bend Center, South Bend, IN 46617, USA; yangao@iu.edu; 2 Department of Biological Sciences, University of Notre Dame, Notre Dame, IN 46556, USA; 3 Central Arkansas Veteran’s Healthcare System, Little Rock, AR 72205, USA; Karl.Straub@va.gov; 4 Departments of Medicine and Biochemistry, University of Arkansas for Medical Sciences, Little Rock, AR 72202, USA

**Keywords:** reactive sulfur species, reactive oxygen species, antioxidants

## Abstract

We have shown that autoxidized polyphenolic nutraceuticals oxidize H_2_S to polysulfides and thiosulfate and this may convey their cytoprotective effects. Polyphenol reactivity is largely attributed to the B ring, which is usually a form of hydroxyquinone (HQ). Here, we examine the effects of HQs on sulfur metabolism using H_2_S- and polysulfide-specific fluorophores (AzMC and SSP4, respectively) and thiosulfate sensitive silver nanoparticles (AgNP). In buffer, 1,4-dihydroxybenzene (1,4-DB), 1,4-benzoquinone (1,4-BQ), pyrogallol (PG) and gallic acid (GA) oxidized H_2_S to polysulfides and thiosulfate, whereas 1,2-DB, 1,3-DB, 1,2-dihydroxy,3,4-benzoquinone and shikimic acid did not. In addition, 1,4-DB, 1,4-BQ, PG and GA also increased polysulfide production in HEK293 cells. In buffer, H_2_S oxidation by 1,4-DB was oxygen-dependent, partially inhibited by tempol and trolox, and absorbance spectra were consistent with redox cycling between HQ autoxidation and H_2_S-mediated reduction. Neither 1,2-DB, 1,3-DB, 1,4-DB nor 1,4-BQ reduced polysulfides to H_2_S in either 21% or 0% oxygen. Epinephrine and norepinephrine also oxidized H_2_S to polysulfides and thiosulfate; dopamine and tyrosine were ineffective. Polyphenones were also examined, but only 2,5-dihydroxy- and 2,3,4-trihydroxybenzophenones oxidized H_2_S. These results show that H_2_S is readily oxidized by specific hydroxyquinones and quinones, most likely through the formation of a semiquinone radical intermediate derived from either reaction of oxygen with the reduced quinones, or from direct reaction between H_2_S and quinones. We propose that polysulfide production by these reactions contributes to the health-promoting benefits of polyphenolic nutraceuticals.

## 1. Introduction

There is increasing interest in the health benefits of a variety of teas [1]. Green tea and its polyphenolic catechins are especially noteworthy for their anticancer properties [2,3,4,5], protection of the cardiovascular system and associated metabolic diseases [6,7,8] and protection against neurodegenerative diseases [9,10]. The presumed biological efficacy of these polyphenols is attributed to their antioxidant properties and ability to serve as free radical scavengers and quench reactive oxygen species (ROS) [11,12], although they may also form covalent adducts with protein and non-protein thiols [13,14] and may bind to, or sterically interfere with, enzymes or other regulatory proteins [15].

Tea polyphenols may also autoxidize and thereby become pro-oxidants [11,12]. We recently showed that these autoxidized green teas and their catechins catalytically oxidized hydrogen sulfide (H_2_S) to polysulfides (H_2_S_n_; where *n* = 2–5) and other sulfoxides, especially thiosulfate [16]. As these products are themselves potent direct antioxidants [17] and will initiate Nrf2-mediated transcription of antioxidant defenses [18,19], we proposed that many of the health benefits of teas could be attributed to their autoxidation and subsequent effects on cellular sulfur metabolism. The autoxidative step is consistent with the analysis of Forman et al. [20], who argued that free radical scavenging by polyphenols is kinetically unrealistic and that the health benefits of these compounds were due to their autoxidation and subsequent increase in electrophilic signaling.

It is generally accepted that oxidation of polyphenols such as catechin occurs at the B ring hydroquinone, which undergoes a one-electron oxidation to a semiquinone radical or a two-electron oxidation to the quinone. However, while oxygen-mediated oxidation of polyphenol (PhOH) anions is thermodynamically favored, it is kinetically unfavorable [2]. This has been suggested to be overcome by trace-metal catalysis [2], although catechins can also undergo autoxidation by O_2_ in the absence of metal catalysts, albeit slowly [21]. The latter is consistent with our observations of polysulfide production from H_2_S catalyzed by tea catechins [16]. Two or three vicinal hydroxyl (catechol) groups of the B ring render these compounds electron-rich and give catechins their antioxidant function [2,22], and their antioxidant efficiency increases as the number of hydroxyl groups increases [1,2,23]. This also correlates with their ability to oxidize H_2_S [16].

In the present study, we examine the potential contribution of the B ring in H_2_S oxidation to polysulfides because (1) the B ring is the primary catalytic site of nutraceutical polyphenols, (2) these polyphenols have been shown to oxidize H_2_S to polysulfides, and (3) polysulfides are well-known antioxidants and cytoprotective compounds. Here, we examine a number of variously substituted hydroxybenzenes, benzoquinones and other quinoid molecules and we show that 1,4-dihydroxybenzene is readily autoxidized by O_2_, which enables it to subsequently oxidize H_2_S to polysulfides. Neither 1,2 nor 1,3-hydroxybenzenes are readily oxidized, nor do they oxidize H_2_S. Conversely, 1,2,3-trihydroxybenzenes readily oxidize H_2_S. Like monobenzenes, 2,5-dihydroxybenzophenone and 2,3,4-trihydroxybenzophenone readily oxidize whereas other combinations of hydroxyl groups spread across both benzene rings do not. Similar effects on sulfur metabolism were observed when these molecules were added to HEK293 cells. Catecholamines having 1,2-hydroxybenzene also oxidize H_2_S, suggesting that side groups can further modify reactivity and extend the suite of H_2_S-oxidizing phenols. These results provide the chemical basis for nutraceutical polyphenol metabolism of sulfur and may explain some of their biological attributes.

## 2. Results

### 2.1. H_2_S Oxidation by Quinones and Hydroquinones

#### 2.1.1. Polysulfide Production

**Figure 1:**
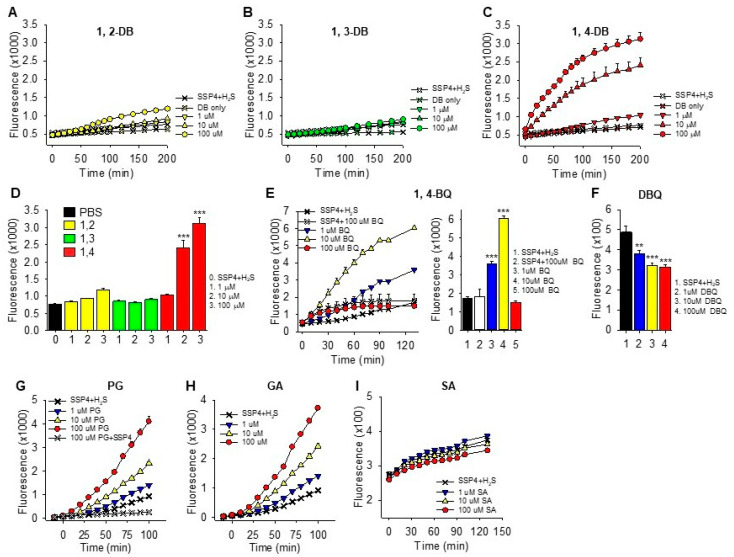
Effects of *ortho* (1,2), *meta* (1,3) and *para* (1,4) dihydroxybenzenes (DB), (**A**–**C**, respectively, 200 min summary in **D**), benzoquinone (BQ; **E**), 2,5-dihydroxy-1,4-benzoqinone (DBQ; **F**), pyrogallol (PG; **G**), Gallic acid (GA; **H**) and shikimic acid (SH; **I**) on polysulfide formation (SSP4 fluorescence) from 300 μM H_2_S in PBS. Polysulfides were concentration-dependently formed from 10 and 100 μM 1,4-DB; neither 1,2-DB nor 1,3-DB significantly increased fluorescence. Polysulfides were also produced by 1 and 10 μM *p*-benzoquinone (BQ) but SSP4 fluorescence did not increase in the presence of 100 μM BQ. DBQ slightly but significantly decreased SSP4 fluorescence. Both pyrogallol and gallic acid concentration-dependently increased polysulfides; shikimic acid did not. Compounds were added in the order SSP4, DB, BQ or DBQ then H_2_S. Mean + SE, *n* = 4 wells per treatment; **, *p* < 0.01; ***, *p* < 0.001.

The effects of *ortho* (1,2), *meta* (1,3) and *para* (1,4) dihydroxybenzenes (DB; also known as, catechol, resorcinol and hydroquinone, respectively), *p*-benzoquinone (1,4-BQ), 2,5-dihydroxy-1,4-benzoqinone (DBQ), pyrogallol (PG), gallic acid (GA) and shikimic acid (SA) on polysulfide formation (SSP4 fluorescence) from 300 µM H_2_S in PBS are shown in Figure 1. Polysulfides were concentration-dependently formed from 10 and 100 µM 1,4-DB. Neither 1,2-DB nor 1,3-DB significantly increased polysulfide production, although there appeared to be a slight effect of 1,2-DB.

Benzoquinone concentration-dependently increased polysulfide production at 1 and 10 μM, whereas 100 μM 1,4-BQ was not significantly different from the control. The apparent inhibitory effect of 100 μM 1,4-BQ could be due to optical interference with the SSP4 Ex/Em, which we have shown for porphyrins [24], or 1,4-BQ interfering with the reaction between the polysulfide and SSP4. As we did not observe a noticeable optical interference with 1,4 DB, the latter option seemed more reasonable. To examine this more thoroughly, we then added two of the three reactants and incubated them for 5, 10 or 30 min before adding the third. As shown in Supplemental Figure S1, the rate of polysulfide formation was greatest when 1,4-BQ and H_2_S were added prior to SSP4, whereas adding 1,4-BQ to SSP4 prior to H_2_S nearly completely prevented the increase in SSP4 fluorescence. Sequential addition of SSP4, H_2_S and 1,4-BQ, which took approximately 1 min between compounds, was sufficient to overcome the inhibitory effect of 1,4-BQ. These results show that 100 μM 1,4-BQ inhibits SSP4 fluorescence when added to SSP4 prior to the addition of H_2_S and this was avoided in subsequent experiments. This inhibitory effect was not observed with 1 or 10 μM 1,4-BQ. The rapid increase in SSP4 fluorescence observed when 1,4-BQ and H_2_S were added 5 min prior to SSP4 also suggests that this reaction is quite rapid, possibly being essentially completed in 5 min or less.

Surprisingly, 2,5-dihydroxy-1,4-benzoqinone (DBQ) did not produce polysulfides from H_2_S. In fact, SSP4 fluorescence was significantly decreased (~35%) by DBQ. The nature of this inhibition was not examined further, although DBQ is known to be a chemically stable molecule and may not react with oxygen or sulfide [25].

##### Mechanism of Quinone-Catalyzed Polysulfide Production

It is unlikely that hydroxybenzenes (HB) will react directly with H_2_S as both molecules are in their reduced state. This suggests that the former are initially autoxidized prior to reacting with H_2_S. To examine this possibility, we used 1,4-DB and PG to examine the O_2_ dependency of H_2_S oxidation, the absorbance spectra during autoxidation and H_2_S reduction, the effects of removing ROS produced by O_2_ reduction (superoxide and peroxide) with Cu/Zn superoxide dismutase (SOD). We also examined the effects of free radical and ROS scavengers on H_2_S oxidation by 1,4-DB and PG. In some instances, the oxidized 1,4-BQ was included for comparison. Unfortunately, we could not examine the effects of catalase as it interfered with the reaction between the polysulfides and SSP4.

##### O_2_ Dependency of H_2_S Oxidation

**Figure 2:**
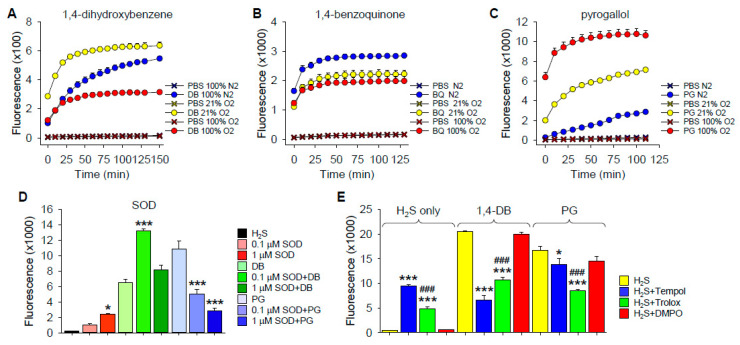
Mechanisms of dihydroxybenzene-catalyzed polysulfide production. (**A**–**C**) Oxygen dependency of H_2_S oxidation to polysulfides (SSP4 fluorescence) by 1,4-dihydroxybenzene (DB, **A**), benzoquinone (BQ, **B**) and pyrogallol (PG, **C**). Buffer containing 100 μM DB, BQ or PG in 3-mL glass vials was bubbled with 100% nitrogen (N_2_), 21% oxygen (21% O_2_) or 100% oxygen (100% O_2_) for 20 min. H_2_S (300 μM) was then added for 10 min, after which 200-μL aliquots were placed into 96-well plates and SSP4 added. With DB, polysulfide production increased between 0 and 21% O_2_ but was decreased by 100% O_2_. Effects of O_2_ on polysulfide production by BQ were minimal, whereas polysulfide production by PG increased with increasing O_2_. Mean + SE, *n* = 4 wells per treatment. (**D**) Effects of superoxide dismutase (SOD) on polysulfide production (SSP4 fluorescence) produced by 100 μM 1,4-dihydroxybenxene (DB) or pyrogallol (PG) oxidation of 300 μM H_2_S in 21% O_2_. SOD, DB and PG were added to wells 10 min prior to H_2_S. SSP4 was added 10 min later and samples counted; results show responses at 130 min. SOD at 0.1 μM did not oxidize H_2_S to polysulfides in buffer, whereas it increased polysulfide production by DB and decreased polysulfide production by PG; 1 μM SOD increased polysulfide production from H_2_S in buffer and decreased polysulfide production by both DB and PG. Mean + SE, *n* = 4 wells; *, *p* < 0.05, ***, *p* < 0.001 compared to like treatment without enzymes. (**E**) Effects of ROS and free radical scavengers, tempol, trolox or DMPO (all 1 mM) on polysulfide production (SSP4 fluorescence) from 300 μM H_2_S alone or H_2_S plus 100 μM 1,4-dihydroxybenzene (DB) or 100 μM pyrogallol (PG). Scavengers and DB or PG were added to wells and allowed to react for 10 min before H_2_S; SSP4 was added 10 min later. Both tempol and trolox increased polysulfide production when added to H_2_S but decreased production when H_2_S was added to either DB or PG; DMPO was ineffective in all conditions. The effect of tempol on polysulfide production from H_2_S and H_2_S plus DB was greater than that of trolox but less than H_2_S plus PG. Mean + SE *n* = 4 wells per treatment; *, *p* < 0.05; ***, *p* < 0.001, significantly different from like control; ###, *p* < 0.001, significant difference between tempol and trolox.

To determine if DB autoxidation was dependent on O_2_, we bubbled buffer with 1,4-DB, 1,4-BQ and PG (PG) for 20–30 min with either 0 or 100% O_2_, or used buffer equilibrated with room air (21% O_2_), then added H_2_S for 10 min before aliquoting samples into the well plates and adding SSP4. As shown in Figure 2A–C, polysulfide production by 1,4-DB was increased between 0 and 21% O_2_ but decreased by 100% O_2_. Polysulfide production by 1,4-BQ was slightly greater at 0% O_2_ but there was no difference between 21 and 100% O_2_. Polysulfide production by PG was strongly dependent on O_2_, progressively increasing with increasing O_2_. These results suggest that autoxidation and subsequent reaction of DB and PG with H_2_S is an O_2_-dependent process. The decrease in polysulfide production by 1,4-DB at 100% O_2_ likely reflects the rapid autoxidation of 1,4-DB to 1,4-BQ and subsequent inhibition of the reaction with SSP4. An alternate explanation is that the polysulfides formed are subsequently oxidized by molecular oxygen and that, at high oxygen concentrations, this reaction becomes predominant. The increase in polysulfide production by 1,4-BQ in anoxia may indicate a small amount of reduction of 1,4-BQ to 1,4-DB.

##### Effects of Superoxide Dismutase

Oxygen-dependent oxidation of hydroxybenzenes has been shown to produce both superoxide and peroxide [22,26,27,28,29]. In order to determine if superoxide affected H_2_S oxidation, we added H_2_S to either 1,4-DB or PG in the presence of superoxide dismutase (SOD) in buffer at 21% O_2_. As we have previously shown that SOD concentration-dependently oxidizes H_2_S to polysulfides in O_2_ [30], we selected enzyme concentrations below or close to their respective thresholds for H_2_S oxidation and evaluated their effects on polysulfide production (Figure 2D).

The low concentration of SOD (0.1 μM) by itself did not significantly increase polysulfide production from H_2_S, whereas it doubled 1,4-DB-mediated polysulfide production and halved the amount of polysulfides produced by PG (Figure 2D). One μM SOD by itself increased polysulfide production in buffer but did not significantly affect polysulfides produced by 1,4-DB and further decreased PG-mediated polysulfide production. These results confirm our previous findings that SOD by itself oxidizes H_2_S to polysulfides and they also show that SOD has distinct effects on polysulfide production by 1,4-DB and PG. This also suggests that H_2_S oxidation by 1,4-DB and PG is mediated by different mechanisms.

##### Effects of ROS/Redox Scavengers on H_2_S Oxidation by 1,4-DB and Pyrogallol

To examine the potential contribution of ROS to H_2_S oxidation by 1,4-DB or PG, we incubated these compounds with the superoxide scavenger and superoxide dismutase mimetic, 4-hydroxy-2,2,6,6-tetramethylpiperidin-1-oxyl (tempol), the free radical scavenger and water-soluble vitamin E analog, 6-hydroxy-2,5,7,8-tetramethylchroman-2-carboxylic acid (trolox), and the nitric oxide spin trap, 5,5-dimethyl-1-pyrroline N-oxide (DMPO). As shown in Figure 2E, both tempol and trolox increased polysulfide production when added to H_2_S but decreased production when H_2_S was added to either 1,4-DB or PG. The effect of tempol on polysulfide production from H_2_S alone or H_2_S plus PG was significantly greater than that of trolox but less than trolox with H_2_S plus 1,4-DB, suggesting differences in 1,4-DB and PG reactions. DMPO was ineffective in all conditions.

Tempol inhibited polysulfide production with 1,4-DB by 67% and the SSP4 fluorescence produced by H_2_S plus 1,4-DB was slightly less than fluorescence produced by tempol with H_2_S alone, suggesting that tempol completely inhibited polysulfide production from 1,4-DB. Conversely, tempol only decreased polysulfide production with PG by 17%, again suggesting that the pathway of oxidation in these two hydroquinones is different. The inhibitory effect of trolox was essentially the same for 1,4-DB and PG (48 and 50%, respectively), indicating that trolox was effective in reducing the formation of polysulfides and that the free radical semiquinone intermediates are important reactants with H_2_S. Thus, reducing semiquinone concentrations by trolox reduces the amount of semiquinones available for reaction with H_2_S and therefore reduces the polysulfide formation.

#### 2.1.2. Thiosulfate Production

We have previously demonstrated that green tea polyphenols oxidize H_2_S to thiosulfate in addition to polysulfides [16]. Here, we used the AgNP assay to examine thiosulfate production by various benzenes.

**Figure 3:**
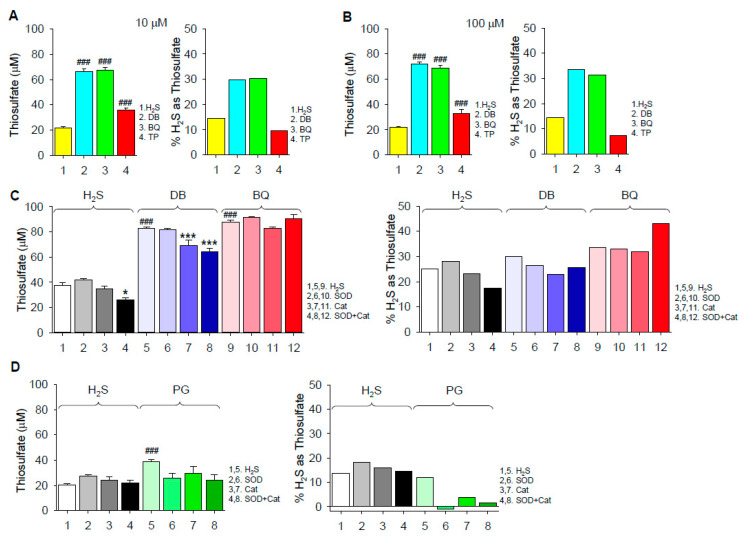
(**A**,**B**) Thiosulfate production from 300 μM H_2_S autoxidation and H_2_S combined with either 10 (**A**) or 100 μM (**B**) 1,4-dihydroxybenzene (DB), 1,4-benzoquinone (BQ) and 2,3,4-trihydroxybenzophenone (TP). Left panels in (**A**) and (**B**) show thiosulfate concentration, right panels show percent H_2_S converted to thiosulfate assuming 2 moles H_2_S per mole of thiosulfate. Percent conversion for DB, BQ and TP is corrected for H_2_S conversion. (**C**,**D**) Effects of 0.1 μM superoxide dismutase (SOD) and 0.1 μM catalase (Cat), alone and in combination, on autoxidation of 300 μM H_2_S, and H_2_S with 10 μM DB and 10 μM BQ (**C**) or 10 μM pyrogallol (PG; **D**). Percent conversion by DB, BQ and PG is corrected for H_2_S autoxidation. SOD did not affect thiosulfate produced by H_2_S autoxidation or H_2_S plus DB, BQ or PG, whereas Cat slightly decreased the effect of DB with or without SOD. Mean + SE; *n* = 8 wells per treatment; ###, *p* < 0.001 compared to H_2_S control; *, *p* < 0.05; ***, *p* < 0.001 compared to respective control.

Thiosulfate was produced by autoxidation of 300 μM H_2_S in the absence of quinones and it was further increased by 10 μM and 100 μM 1,4-DB or 1,4-BQ (Figure 3A,B). Increasing the concentration of the quinones from 10 to 100 μM only slightly increased thiosulfate production, suggesting that the available H_2_S or O_2_ was exhausted by the combined production of thiosulfate, polysulfides and possibly other oxidized sulfur molecules. Approximately 30% of the initial H_2_S was converted to thiosulfate by both DB and BQ. SOD did not affect thiosulfate produced by H_2_S autoxidation or H_2_S plus 1,4-DB or 1,4-BQ, whereas Cat slightly decreased the effect of 1,4-DB with or without SOD (Figure 3C,D). However, when the percent conversion of H_2_S to thiosulfate was corrected for H_2_S autoxidation, it was evident that both SOD and Cat inhibited the effect of PG. These results suggest that H_2_S oxidation to thiosulfate by 1,4-DB and 1,4-BQ is independent of concomitant formation of superoxide and only 1,4-DB is slightly (< 20%) dependent on hydrogen peroxide. They also suggest that H_2_S oxidation by 1,4-DB and 1,4-BQ is predominantly mediated by the common formation of the semiquinone radical, whereas PG-catalyzed H_2_S oxidation is a different process, likely mediated by hydrogen peroxide.

#### 2.1.3. Absorbance Spectra of Hydroxybenzene/Benzoquinone Redox Reactions

**Figure 4:**
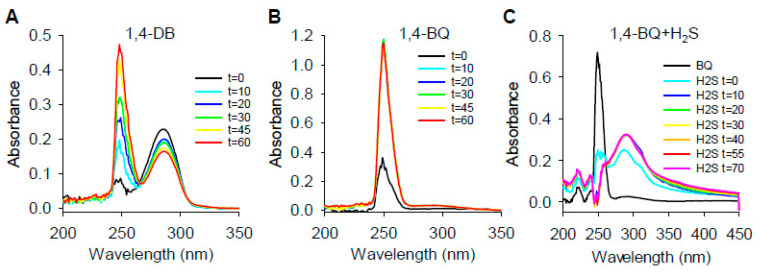
Effects of redox reactions on the absorption spectra of 100 μM 1,4-DB (DB) and 100 μM 1,4-BQ (BQ) in room air (21% O_2_). (**A**) DB was autoxidized to BQ in 21% O_2_ as shown by a decrease the absorption peak at 289 nm and an increase in absorption at 249 nm. (**B**) The BQ absorption peak at 249 nm was unaffected by O_2_, whereas addition of 100 μM H_2_S to BQ (**C**) rapidly decreased the absorbance peak at 249 nm and increased absorbance at 289 nm, indicative of reduction of BQ to DB.

Firstly, 1,4-DB and 1,4-BQ have distinct absorbance peaks at ~290 and ~250 nm, respectively [31], which we confirmed (Figure 4A,B). To determine if 1,4-DB is autoxidized, we monitored the absorbance spectra of 1,4-DB in 21% O_2_ over time and observed a decrease in the 289-nm peak and a concomitant increase in the 249-nm peak, indicating that, indeed, 1,4-DB is autoxidized to 1,4-BQ (Figure 4A). Furthermore, adding H_2_S to 1,4-BQ decreased the 249-nm peak and increased the 289-nm peak (Figure 4C), showing that 1,4-BQ can be reduced to 1,4-DB by H_2_S. These results indicate that 1,4-DB is autoxidized to 1,4-BQ in the presence of O_2_ and that H_2_S effectively regenerates 1,4-DB by reducing 1,4-BQ. The latter process is likely achieved by concomitant oxidation of H_2_S to polysulfides and thiosulfate. A similar reduction of 1,4-BQ to DB by glutathione (GSH) was observed by Su et al. [31], confirming that 1,4-BQ is reduced by other thiols.

To examine the interaction between O_2_ and H_2_S on absorption spectra of 1,4-DB, we bubbled buffer with 100% N_2_, 21% O_2_ or 100% O_2_ for 20 min prior to the addition of the compounds. As shown in Supplemental Figure S2, in 100% N_2_, there was only a slight decrease in absorption at 289 nm, indicating very little 1,4-DB oxidation, although a slight 1,4-BQ peak was also observed. Addition of 100 μM H_2_S reduced most 1,4-BQ to 1,4-DB. As the O_2_ tension increased, there was a concomitant increase in 1,4-DB oxidation, as seen by a decrease in the 289-nm peak and an increase in the 249-nm peak. Again, 1,4-BQ was reduced to 1,4-DB after the addition of H_2_S. These results provide additional evidence that O_2_ is involved in 1,4-DB autoxidation and that this is a reversible process.

Our results also show that neither 1,2- nor 1,3-dihydroxybenzene (1,2-DB and 1,3-DB, respectively) oxidize H_2_S (Figure 1), implying that they are not appreciably autoxidized by O_2_. Knowing that 1,2-DB and 1,2-benzoquinone (1,2-BQ) exhibit absorption maxima at 276 and 389 nm, respectively [32], we examined the absorption spectra of 1,2-DB in room air (21% O_2_) and in the presence of CuCl_2_ to determine if it was autoxidized or could be oxidized in the presence of other oxidants. As shown in Supplemental Figure S3A–D, the absorption peak for the 1,2-DB was 273 nm and, although it decreased somewhat after 60 min, there was no evidence for a peak corresponding to 1,2-BQ, nor was there any evidence of 1,2-BQ peak produced when CuCl_2_ was added. However, CuCl_2_ appeared to concentration-dependently increase absorbance at 249 nm. Although this suggests that some of the 1,2-DB was converted to 1,4-DB, there was no evidence that 1,4-DB was then autoxidized to 1,4-BQ, which would have been expected as CuCl_2_ readily oxidized 1,4-DB to 1,4-BQ (Supplemental Figure S3E). Therefore, we conclude that the 249-nm peak is not due to the appearance of 1,4-DB, which is very unlikely, but due to an unidentified molecule. We also found no evidence for autoxidation of 1,3-DB (Supplemental Figure S3F). Collectively, these results indicate that the oxidation of a dihydroxybenzene to the semiquinone and then to the quinone is a pathway for H_2_S oxidation, and of the three DB examined, this only occurs with 1,4-DB.

The stoichiometry of 1,4-BQ reduction by H_2_S and the time course for this reaction was then examined (Supplemental Figure S4). The 1,4-BQ absorption maximum at 249 nm was concentration-dependently reduced by H_2_S and it was essentially eliminated at 100 μM H_2_S. This coincided with an H_2_S concentration-dependent increase in absorbance at 289 nm, consistent with the formation of 1,4-DB. Furthermore, maximum absorbance at 289 nm (~0.3 absorbance units) suggests that all the 1,4-BQ was converted to 1,4-DB (Figure 3). Spectral changes of both 249 and 289 nm were completed within 10 min when H_2_S was 100 μM or greater. It is evident from Supplemental Figure S4 that more than half of the 1,4-BQ is reduced and 1,4-DB formed by 50 μM H_2_S, leading to the conclusion that the reaction is more complex than can be explained by a simple stoichiometric relationship.

If one considers the fully reduced state at 249 nm to be approximately 0.25 OD, then the change in OD from oxidized to reduced is approximately 0.93. Using this metric, the change by the addition of 25 micromolar H_2_S is approximately 0.15 OD, and on the addition of 50 micromolar H_2_S, it is approximately 0.55 OD. Thus, doubling the concentration of H_2_S causes a quadrupling of the reduction of BQ. If the reaction is reversible, then the reduction of BQ to DB requires two moles of H_2_S for every mole of BQ reduced, which is to be expected if the reduction involves two one-electron steps with one electron being provided by H_2_S at each step.

Unlike 1,2- and 1,3-DB, it has been shown that PG is readily autoxidized to purpurogallin (2,3,4,6-tetrahydroxy-5H-benzocycloheptene-5) in the presence of O_2_ and this can be observed by a red shift in the absorbance peak from ~275 to ~320 nm. It may then undergo a second O_2_-dependent oxidation to a dianion of purpurogallin quinone (absorbance peak at 600 nM), and a final transformation, possibly requiring H_2_O_2_, to a product with an absorption peak at 440 nm by > 100 min [33]. Our spectral analysis of PG autoxidation in 21% O_2_ (Supplemental Figure S5A) also showed an increase in absorbance at 320 nm, indicative of the production of purpurogallin. However, the 320-nm peak was unaffected by H_2_S. Although we did not extensively examine all spectral changes, we did not observe an increase in absorbance at 440 nm. The oxidation of PG to purpurogallin is an irreversible set of reactions [34] which involves an intermediate, 3-hydroxy o-benzoquinone [35], or the purpurogalloquinone [33], and it is one or both of these compounds which most likely reacts with H_2_S.

Gallic acid (GA) can be oxidized in the presence of trace transition metals to a semiquinone radical and then to *o*-quinone derivatives with an increase in an absorbance peak at 420 nm, similar to that of PG [36]. While we observed an absorbance peak at 255 nm, we did not observe any peak appearing at 420 nm after incubation of GA in buffer at 21% O_2_, nor did this peak change after H_2_S was added (Supplemental Figure S5B). It has been reported that GA does not autoxidize below pH 8.5 [37]. These results suggest that either PG and GA respond differently to O_2_ and H_2_S, or that H_2_S rapidly reacts with the quinone intermediates which are transient and of low concentration and not seen by spectrometry and that, once the final products are formed, they cannot be reduced by H_2_S.

#### 2.1.4. Hydroxybenzene and Benzoquinone Comproportionation, Semiquinone Formation and Polysulfide Production

It has been reported that when 1,4-DB and 1,4-BQ are added together, they will comproportionate and form a dimer (two spin-paired semiquinone radicals), commonly called a quinhydrone, that is relatively stable [38,39]. To examine if this was the case under our experimental conditions, we obtained the spectrum of either 100 μM 1,4-DB or 100 μM 1,4-BQ while varying the concentration of the other (Supplemental Figures S6 and S7, respectively). As reported above, in the absence of 1,4-BQ, the spectrum for 1,4-DB exhibited increasing absorbance at 249 nm, indicative of oxidation to 1,4-BQ, while the 1,4-DB peak at 289 nm slightly decreased (Supplemental Figure S6). Increasing 1,4-BQ concentration from 0.1 to 10 μM had little effect on either peak except to hasten the increase in the peak at 289 nm. Addition of 100 μM 1,4-BQ immediately produced a strong 1,4-BQ spectrum that rapidly decayed. With the exception of this transient 1,4-BQ peak, the rate of change of the 1,4-DB peak appeared to be little affected by 1,4-BQ. Similarly, the 1,4-BQ peak at 249 nm was not appreciably affected by 0.1–100 μM 1,4-DB, nor was there any appreciable accumulation of 1,4-DB until the 1,4-DB concentration reached 100 μM (Supplemental Figure S7).

To examine comproportionation with respect to H_2_S oxidation to polysulfides (SSP4 fluorescence), we added increasing concentrations of 1,4-BQ to 100 μM 1,4-DB and observed that 0.1 and 1 µM 1,4-BQ did not increase the rate of 1,4-DB autoxidation, whereas 10 μM 1,4-BQ decreased it (Supplemental Figure S8). The lack of effect at low 1,4-BQ concentrations was likely due to some contamination of our 1,4-DB sample with a small amount of 1,4-BQ, which is common [38] and supported by the appearance of a small peak at 249 nm in our sample (Figure 4A). The decreased response with 10 μM 1,4-BQ could be due to 1,4-BQ interfering with the polysulfide–SSP4 reaction, although this seems unlikely as the SSP4 was added 10 min after H_2_S and, by this time, 300 μM H_2_S should have reduced most, if not all, 1,4-BQ to 1,4-DB (Figure 4C). Reportedly, 1,4-DB will autoxidize very slowly until 1,4-BQ is formed; then, after this slow induction phase, the reaction speeds up and it becomes independent of 1,4-BQ concentration and our experiments confirm this. Rathore and Chandel [27] have confirmed that the oxidation of H_2_S by oxygen is catalyzed by 1,4-DB, which is just another way of looking at the reaction of 1,4-DB with H_2_S. The initial reaction is the oxidation of 1,4-DB, not H_2_S. However, our results suggest that if comproportionation does occur, it does not do so in the timescale that would generate sufficient additional semiquinone radicals to significantly increase the rate of H_2_S oxidation to polysulfides.

#### 2.1.5. Hydroxybenzenes and Benzoquinone as Polysulfide Reductants

To determine if 1,4-DB or its oxidized counterpart, 1,4-BQ, would regenerate H_2_S from polysulfides, we added them to the mixed polysulfide, K_2_S_n_, and measured H_2_S production with the H_2_S fluorophore, AzMC, in buffer bubbled with 21% O_2_ or 100% N_2_. There was no evidence that any of these compounds produced H_2_S from polysulfides in either oxic or anoxic conditions (Supplemental Figure S9). This suggests that they may not function as reductants in cellular sulfur metabolism. The significant decrease in AzMC fluorescence produced by 100 μM 1,4-BQ is consistent with the above observations that 1,4-BQ interferes with the reaction between these fluorophores and sulfur compounds.

#### 2.1.6. Effects of Dihydroxybenzene and *p*-Benzoquinone on Cellular Sulfur Metabolism

**Figure 5:**
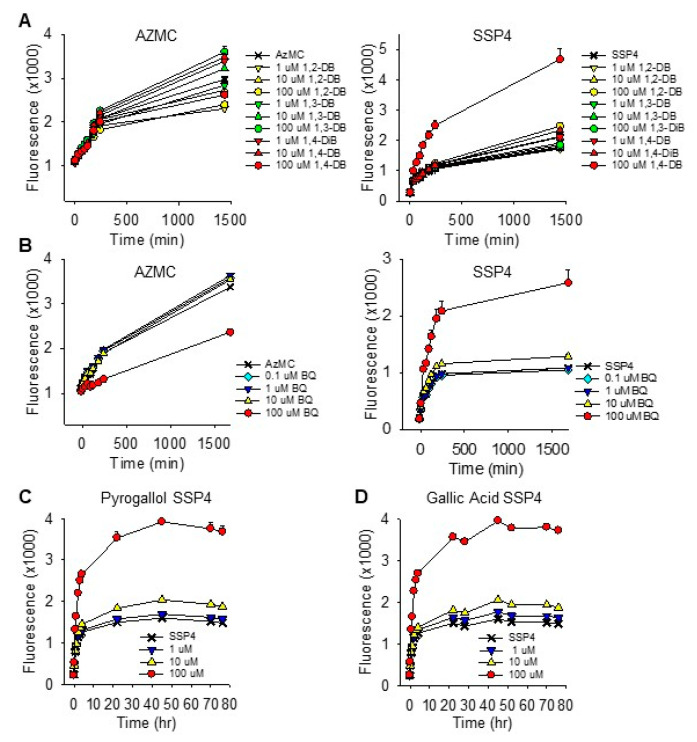
Effects of 1,4-DB (DB; **A**,**B**) and 1,4-BQ (BQ; **B**,**C**) on H_2_S and polysulfide production (AzMC and SSP4 fluorescence, respectively) in HEK293. AzMC fluorescence was not consistently affected by DB, but it was significantly (*p* < 0.001) decreased by BQ. Only 100 μM 1,4-DB and 100 μM BQ increased SSP4 fluorescence. (**C**,**D**) Effects of pyrogallol and gallic acid, respectively, on polysulfide (SSP4 fluorescence) in HEK cells. Both compounds at 100 μM increased polysulfide production after 2 h (*p* < 0.001). Mean + SE, *n* = 8 wells per treatment.

The effects of 1,4-dihydroxybenzene (1,4-DB) and 1,4-benzoquinone (1,4-BQ) on H_2_S and polysulfide production in HEK293 cells are shown in Figure 5A,B. H_2_S production (AzMC fluorescence) was not affected by 1,4-DB but decreased by 100 μM 1,4-BQ. Only 100 μM 1,4-DB and 100 μM 1,4-BQ increased polysulfide production (SSP4 fluorescence) and the initial increase in SSP4 fluorescence appeared to be faster with 1,4-BQ than it was with 1,4-DB. Both PG (C) and GA (D) increased polysulfide production in these cells and this increase was sustained for over 3 days; H_2_S was not examined. These results are generally consistent with their effects on sulfur metabolism in buffer and they suggest that one of the primary effects of these compounds in cells is to increase polysulfide production.

### 2.2. H_2_S Oxidation by Catecholamines

Although catechol (1,2-dihydroxybenzene) did not appear to appreciably oxidize H_2_S, epinephrine, which has hydroxyl groups in the 3,4 position, has been reported to autoxidize to the quinone [40]. The following experiments were conducted to determine if this was sufficient to oxidize H_2_S.

**Figure 6:**
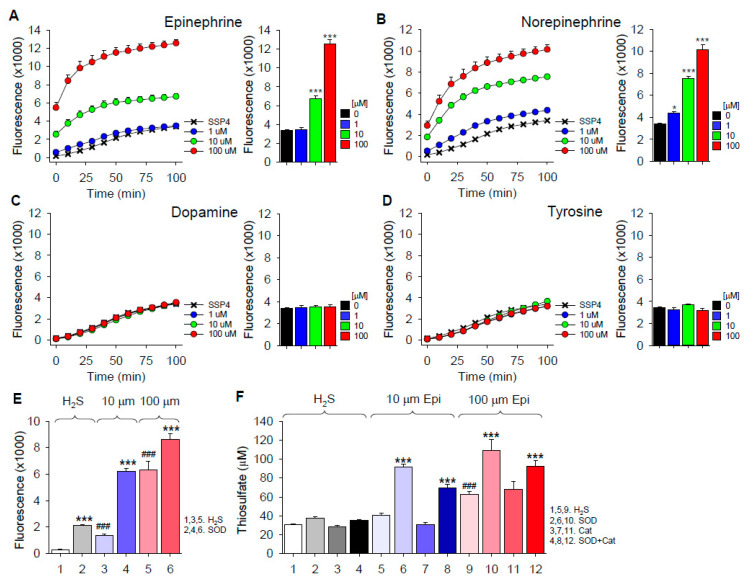
(**A**–**D**) Effects of biogenic amines on polysulfide production (SSP4 fluorescence) from 300 μM H_2_S. Both epinephrine and norepinephrine concentration-dependently increased polysulfide production whereas dopamine and tyrosine did not. (**E**,**F**) Superoxide dismutase (SOD; 0.1 μM) increases polysulfide (SSP4 fluorescence) and thiosulfate (AgNP absorbance) production from 10 and 100 μM epinephrine (Epi) and 300 μM H_2_S, whereas catalase (Cat; 0.1 μM) was ineffective. Mean + SE, *n* = 4 (**A**–**E**) or 8 (**F**) wells per treatment; *, *p* < 0.05; ***, *p* < 0.001 compared to respective control; ###, *p* < 0.001 compared to H_2_S.

Epinephrine, norepinephrine, dopamine and tyrosine were incubated in room air-equilibrated buffer for 30 min; H_2_S was added, followed by SSP4 10 min later. As shown in Figure 6A–D, both epinephrine and norepinephrine concentration-dependently increased polysulfide production from H_2_S, whereas there was no effect of either dopamine or the mono-hydroxy precursor of biogenic amines, tyrosine. These results show that specific side-chain additions to the catechol allow autoxidation, which then can oxidize H_2_S. This contrasts with catechol, which does not oxidize H_2_S to polysulfides (Figure 1).

Given that epinephrine can oxidize H_2_S to polysulfides, we then examined the effects of SOD on polysulfide production and SOD and Cat on thiosulfate production from H_2_S and epinephrine (Figure 6E,F, respectively). Both polysulfide and thiosulfate production were significantly increased by SOD, whereas Cat did not affect thiosulfate production. These results suggest that neither superoxide nor hydrogen peroxide are important in the oxidation of H_2_S to polysulfides or thiosulfate. As both reactions are enhanced by SOD, it is possible that by rapidly removing superoxide, the initial reaction of epinephrine and H_2_S is driven the right.

### 2.3. H_2_S Oxidation by Hydroxybenzophenones

Benzophenones, with two benzene rings separated by a carbonyl group, afforded us the opportunity to examine the effects of broadly distributing the hydroxyl groups on H_2_S oxidation to polysulfides. As shown in Supplemental Figure S10, only 2,5-dihydroxybenzophenone and 2,3,4-trihydroxybenzophenone oxidized H_2_S to polysulfides. Incubation of 2,3,4-trihydroxybenzophenone with H_2_S also produced a small amount of thiosulfate (Figure 3A,B), although the amount produced was considerably less than that produced by either 1,4-DB or 1,4-BQ. These results are generally consistent with our observations with the hydroxybenzenes in that *para*-dihydroxyl and adjacent trihydroxy substitutions oxidize H_2_S. Spreading the hydroxyl groups between the benzenes or substituting methoxy for the 4-hydroxyl group in 1,4,-dihydroxybenzophenone appeared to be unable to generate polysulfides.

## 3. Discussion

Much of the biological activity of nutraceutical polyphenols is attributed to the B ring, which is generally considered to convey the majority of the antioxidant and radical-scavenging effects. However, Forman et al. [20] argued that free radical scavenging by polyphenols is kinetically unrealistic and that the health benefits of polyphenols were due to their autoxidation. This produced an increase in electrophilic signaling, Phase II enzymes and nucleophilic substrates including glutathione, thioredoxin and NADPH. These outcomes would also be anticipated to result from increased polysulfide signaling [41].

The present experiments show that simple but specific hydroxybenzenes can oxidize H_2_S to polysulfides after the former are themselves autoxidized. These results help to explain the mechanism behind the health benefits of many nutraceutical polyphenols; polysulfides produced by the oxidation of H_2_S are potent antioxidants [17], they may scavenge superoxide [42], and through their ability to persulfidate Keap1, they free it from Nrf2, which can then activate nuclear antioxidant response elements [18,19,43,44,45,46]. Our experiments also show that the ability of hydroxybenzenes to oxidize H_2_S varies considerably with the location and distribution of these hydroxyl groups and that the addition of specific side groups can further alter the reactivity of these compounds. Furthermore, we show that biogenic amines, epinephrine and norepinephrine, effectively oxidize H_2_S, suggesting possible additional roles for these signaling molecules.

### 3.1. Specificity of Hydroxyl Groups and Their Positioning

#### 3.1.1. Dihydroxybenzenes

It is clear from these studies that hydroxybenzenes must be first autoxidized in order to oxidize H_2_S and that this depends on the number and relative position of hydroxyl groups. We show that at least two hydroxyl groups are required for autoxidation and that autoxidation requires O_2_. Our results also indicate that with respect to otherwise unsubstituted dihydroxybenzenes, the OH groups must be in the *para* position, i.e., 1,4-DB. We did not observe autoxidation of either *ortho*- or *meta*-hydroxybenzenes, nor did we find that they produced polysulfides from H_2_S over the timescale of our experiments. Our results are somewhat in contradiction to those of Roginsky and Alegria [47], who stated that only the *ortho*-, 1,2-DB, can be autoxidized by O_2_ to the semiquinone radical. They also reported that 1,4-DB was not directly autoxidized by O_2_ but reacts with 1,4-BQ to produce two semiquinone radicals. As the latter comproportionation reaction can proceed with trace amounts of 1,4-BQ, and we did not attempt to remove it from our 1,4-DB stock, it is a plausible event, although in the presence of H_2_S, the reaction appears not to need comproportionation. Because the 1,2-DB does not make polysulfides, it may be that the reaction proceeds to the 1,2-BQ by sequential one-electron reductions of oxygen and oxidation of 1,2-BQ; however, 1,2-BQ is unstable [48]. This then reacts with the starting compound, 1,2-DB [49], to make complex compounds or water to give the 1,2,4 trihydroxybenzene [50]. These reactions are fast and there may not be sufficient time for hydrogen sulfide to react in any significant amount with either the semiquinone or the quinone. The production of superoxide at each step explains why SOD accelerates the oxygen consumption by 1,2-DB, as observed by Roginsky and Alegria. We cannot rule out nucleophilic attack by sulfide on the electropositive 4,5 position [51] of the semiquinone or the quinone [52], which would consume H_2_S but not make inorganic polysulfides. The rapid decay of the 1,2-BQ semiquinone can be prevented and has been stabilized and electron paramagnetic resonance (EPR) spectra taken by using di- or trivalent metal ions to chelate the molecule [53]. Roginsky and Alegria [47] also show that 1,3-DB does not autoxidize, which is consistent with our observations. We also observed that benzophenones exhibited similar specificity for OH groups in the 1,4 position, suggesting that this may be a general feature of these molecules.

#### 3.1.2. Trihydroxybenzenes

Both PG and GA readily oxidize H_2_S to polysulfides, whereas shikimic acid does not, further illustrating the importance of the benzene ring. However, our experiments show that while autoxidation of both 1,4-DB and PG requires O_2_, SOD increases polysulfide production from H_2_S by 1,4-DB while it decreases polysulfide production by PG. These results suggest that different autoxidation reactions/products are involved. Abrash et al. [33] have shown that PG is oxidized in three sequential reactions, first to purpurogallin (2,3,4,6-tetrahydroxy-5H-benzocycloheptene-5) in an O_2_-dependent reaction, possibly requiring H_2_O_2_. They also showed that PG oxidation was inhibited by SOD. Our experiments were consistent with these observations; SOD decreased polysulfide production from H_2_S. While our spectral analysis of PG autoxidation in 21% O_2_ also showed an increase in absorbance at 320 nm, indicative of production of purpurogallin, this peak was unaffected by H_2_S. This suggests that H_2_S oxidation may have been due to superoxide produced during PG oxidation, not purpuogallin. PG-mediated oxidation of H_2_S to thiosulfate was also inhibited by SOD, supporting our contention that this is mediated by ROS and not by oxidized PG.

Similarly, while GA is oxidized to *o*-quinone derivatives with an increase in an absorbance peak at 420 nm, similar to that of PG [36], we did not observe any peak appearing at 420 nm after incubation of GA in buffer at 21% O_2_, nor did this peak change after H_2_S was added. This suggests that H_2_S oxidation by GA and PG is similar. Clearly, these reactions require further study but they do not detract from the primary observation that H_2_S is readily oxidized by 1,2,3-trihydroxybenzenes.

#### 3.1.3. Catecholamines

It is also evident from our studies with catecholamines, which are derivatives of 1,2-DB, that having a substituent in the 4 position can affect the stability and reactivity of the 1,2-DB compounds. In this instance, the catechol can become oxidized [40] and form stable semiquinones, and we observed that when oxidized, both norepinephrine and epinephrine, but not dopamine, oxidize H_2_S to polysulfides; epinephrine also produces thiosulfate, while norepinephrine was not examined. Epinephrine has been shown to undergo a one-electron oxidation, producing a semiquinone and superoxide, which gives rise to a chain oxidation reaction [54]. This would be expected to lead to the high rate of reactivity that we observed with H_2_S. The semiquinones of epinephrine and norepinephrine have been stabilized with metal ions and identified by their EPR spectra [55,56]. On the other hand, upon oxidation, dopamine rapidly forms a semiquinone which undergoes a rapid intramolecular condensation to give a five-membered ring attached to the aromatic benzene ring [57]. It is likely that the intramolecular reactions are so fast that H_2_S cannot compete and therefore no polysulfides are made with dopamine.

The side-chain modifications which stabilize the semiquinone of the parent compounds appear to render a variety of neutraceutical polyphenols susceptible to autoxidation and H_2_S oxidation, which we have shown previously for tea catechins [16]. Although 2,5-dihydroxy-1,4-benzoquinone has *para* hydroxyl groups, it does not oxidize H_2_S because it has exceptional resonance stabilization that renders the molecule relatively unreactive [25].

#### 3.1.4. Hydroxybenzophenones

Our results show that benzophenones, with two benzene rings separated by a carbonyl group, generally have similar structural constraints to oxidize H_2_S as the hydroxybenzenes, with only the 2,5-dihydeoxy and the 2,3,4-trihydroxy forms exhibiting appreciable activity. Spreading these OH groups across two benzene rings appeared to be ineffective.

### 3.2. Mechanism of Polysulfide Formation

Our results show that the effects of SOD on H_2_S oxidation by various quinones are quite variable; SOD increased polysulfide production by 1,4-DB and epinephrine and decreased production from PG. Tempol inhibited polysulfide production from 1,4-DB but only slightly decreased it from PG, whereas trolox was equipotent in partially inhibiting H_2_S oxidation by both compounds. These results suggest that H_2_S oxidation by these compounds occurs via a variety of pathways. Although a detailed examination of these pathways is beyond the scope of the present study, several mechanisms are proposed.

#### 3.2.1. Polysulfide Production by 1,4-Dihydroxybenzene

In the following paragraphs, the proposed mechanism for polysulfide production catalyzed by 1,4-DB is provided. While not examined in detail, it is likely that the mechanism catalyzed by epinephrine is similar.

There is considerable evidence that both superoxide (O_2_^•−^) and semiquinone radicals (1,4-SQ^•−^) are produced in the autoxidation of 1,4-DB (Equation (1); [38]).

1,4-DB + O_2_ <–> O_2_^•−^ + 1,4-SQ^•−^ + 2H^+^ (1)

Either one of these radicals, or both, could then oxidize H_2_S, or the HS anion (HS^−^), to a thiyl radical (HS^•^; Equations (2) and (3));

H_2_S + O_2_^•−^ +H^+^ –> HS^•^ + H_2_O_2_ (2)

HS^−^ + SQ_2_^•−^ –> HS^•^ + 1,4-BQ (3)

and two thiyl radicals would then spontaneously combine to form the persulfide (H_2_S_2_; Equation (4));

2HS^•^ –> H_2_S_2_ (4)

Alternatively, one thiyl radical reacts with one hydrosulfide ion (HS^−^; Equation (5));

HS^•^ + HS^−^ –> H_2_S_2_^•−^ (5)

and the dihydrodisulfide radical anion reacts with O_2_, producing the polysulfide and superoxide anion radical (Equation (6));

H_2_S_2_^•−^ + O_2_ –> H_2_S_2_ + O_2_^•−^ (6)

The question becomes which radical, the semiquinone or superoxide, is responsible for oxidizing H_2_S? Are both involved, or is the process a two-electron oxidation with *p*-quinone as the oxidant? Alternatively, do the hydroquinone and quinone engage in a comproportionation cycle that generates reactive semiquinone radicals until the reactants are exhausted?

Although H_2_S is readily oxidized by superoxide [42], our observations that SOD increased polysulfide production from 1,4-DB and epinephrine suggest that superoxide plays a minimal, if any, role in polysulfide production by these two compounds. Rather, by removing superoxide, SOD promotes autoxidation, which is otherwise kinetically unfavorable [39]. This also appears to be the case for thiosulfate production by epinephrine. The nearly complete elimination of the 249-nm absorbance peak of 100 μM 1,4-BQ by 100 μM H_2_S, with no further increase in the peak at 298 nm with 150–200 μM H_2_S, also suggests that H_2_S produces a one-electron reduction of 1,4-BQ to the semiquinone radical without superoxide production. However, SOD may also function as semiquinone:superoxide oxidoreductase in a comproportionation reaction [38], but we have no evidence that this occurs.

Our results also suggest that comproportionation is not involved. The net reaction (Equations (1), (3) and (5)) will consume two moles of O_2_, two moles of H_2_S and make one mole of H_2_S_2_ and two moles of superoxide, while not consuming any net amount of 1,4-DB. The catalytic function of the hydroquinone is supported by our observations that the amount of H_2_S consumed greatly exceeds the amount of 1,4-DB (see Section 3.4. Extent of H_2_S Oxidation by 1,4-Dihydroxybenzene, 1,4-Benzoquinone, Pyrogallol and Epinephrine). Thus, comproportionation of the quinones is not needed to have a catalytic cycle. It also appears from our results that oxidation of H_2_S by a quinone is a different process.

Since 1,3 hydroquinones cannot make a stable semiquinone, no reaction with H_2_S is seen. The same is true for shikimic acid. The 1,2 hydroxybenzene makes a transient semiquinone but reacts so fast that H_2_S cannot compete. Dopamine has a similar fast reaction of the semiquinone which does not allow H_2_S to compete. All the other compounds form a semiquinone if they are to react with H_2_S.

#### 3.2.2. Polysulfide Production by 1,4-Benzoquinone

We propose that if the starting compound is a quinone, then it will oxidize H_2_S, producing a thiyl radical and the semiquinone, the latter reacting with a second H_2_S to form a second thiyl radical and the hydroquinone (Equations (7) and (8)):

HS^−^ + 1,4-BQ –> HS^•^ + 1,4-SQ^•−^ + H^+^ (7)

HS^−^ + 1,4-SQ^•−^ +H^+^ –> HS^•^ + 1,4-DB (8)

These reactions do not produce superoxide and the reaction will slow down when all the quinone is completely oxidized and only resume after some of the hydroquinone is autoxidized. This is shown in Figure 2B, where polysulfide production from the quinone is essentially oxygen-independent and it plateaus rather quickly. Conversely, polysulfide production from the hydroquinone is oxygen-dependent, fails to plateau over the course of the experiment and produces more polysulfides than the quinone (Figure 2B). In 100% oxygen, the rate and extent of H_2_S oxidation by the hydroquinone (Figure 2A) is essentially identical to H_2_S oxidation by the quinone (Figure 2B), which would be expected if all, or nearly all, of the hydroquinone is autoxidized to the quinone prior to the reaction with H_2_S.

### 3.3. Mechanism of Thiosulfate Formation

In addition to polysulfides, 1,4-DB, 1,4-BQ, 2,3,4-trihydroxybenzophenone, PG and epinephrine oxidize H_2_S to thiosulfate. Pyrogallol was the only compound where thiosulfate production was inhibited by catalase, suggesting that this is a different reaction, which also appeared to be the case for polysulfide production. SOD also increased thiosulfate production by epinephrine, decreased it from PG, but had no effect on 1,4-DB or 1,4-BQ.

The relative insensitivity of either 1,4-DB-, 1,4-BQ- or epinephrine-catalyzed thiosulfate production to catalase is consistent with the involvement of a semiquinone radical that oxidizes H_2_S to the thiyl radical, as we propose for polysulfide production. While we did not attempt to further characterize this reaction, it is likely that it involves a reaction between a thiyl radical and a hydrosulfide anion to form a persulfide radical (Equation (9));

HS^•^ + HS^−^ –> HS_2_^•−^ + H^+^ (9)

and subsequent reaction with oxygen (Equation (10));

HS_2_^•−^ + O_2_ + H^+^ –> H_2_S_2_ + O_2_^•−^ (10)

and then oxidation of the polysulfide to thiosulfate (Equation (11));

H_2_S_2_ + 3/2O_2_ –> S_2_O_3_^2−^ + 2H^+^ (11)

with the partial reactions being more complicated and involving several sulfur–oxygen reactive and short-lived intermediates.

### 3.4. Extent of H_2_S Oxidation by 1,4-Dihydroxybenzene, 1,4-Benzoquinone, Pyrogallol and Epinephrine

We can estimate the percent oxidation of 300 μM H_2_S to polysulfides and thiosulfate based on previous SSP4-H_2_S_2_ concentration–response curves [58] and SSP4 fluorescence and on the concentration of thiosulfate produced from 300 μM H_2_S. Polysulfides and thiosulfate sulfur accounted for approximately 20 and 25% of the H_2_S oxidized by 1,4-DB, 5 and 25% of the H_2_S oxidized by 1,4-BQ, 33 and 17% of the H_2_S oxidized by PG and 40 and 45% of the H_2_S oxidized by epinephrine. Autoxidation of H_2_S can also account for 10–35% of the H_2_S consumption. While these estimates are only approximate, they not only show that these compounds can produce substantial amounts of polyphenols and thiosulfate, but they also indicate that anywhere from 20 to 40% of the initial H_2_S is unaccounted for. The identities of these compounds remain to be determined, as well as whether or not they are biologically active.

### 3.5. Other Possible Cytoprotective Effects

Many quinones and semiquinones, including those of the catecholamines, are known to be toxic [59,60,61,62,63,64,65,66]. The ability of H_2_S to reduce them back to the hydroquinones, even at the expense of forming superoxide and hydrogen peroxide, may be important in cellular protection against reactive intermediates. In addition, polysulfides are produced and the polysulfides are very good antioxidants and they also signal the cell to increase its antioxidant defenses. The net effect may be that H_2_S protects against the toxic quinones formed by autoxidation with little cellular damage as long as there is adequate SOD. Conversely, it is an intriguing possibility that the catecholamines might be involved in regulating cellular sulfur metabolism. Clearly, additional work is required.

## 4. Methods

### 4.1. H_2_S and Polysulfide Measurements in Buffer

Compounds of interest were aliquoted into 96-well plates and the wells covered with tape to reduce H_2_S volatilization. Fluorescence was measured with a SpectraMax M5e plate reader (Molecular Devices, Sunnyvale, CA, USA). Excitation/emission (Ex/Em) wavelengths for 7-azido-4-methylcoumarin (AzMC) and 3′,6′-Di(O-thiosalicyl)fluorescein (SSP4) were 365/450 and 482/515 nm, respectively, per manufacturer’s recommendations. Previous studies have shown that these fluorophores have sufficient specificity relative to other sulfur compounds and reactive oxygen and nitrogen species (ROS and RNS, respectively) to effectively identify H_2_S (AzMC) and per- and polysulfides (H_2_S_2_ and H_2_S_n_ where *n* = 3–7) [67,68,69].

### 4.2. Polyphenol Absorbance Spectrum

Absorbance spectra were done on the spectraMax plate reader between 200 and 450 nm. The absorbance of phosphate buffer was subtracted from experimental scans.

### 4.3. Thiosulfate Measurement in Buffer

Thiosulfate was measured with silver nanoparticles (AgNPs) as previously described [70]. Briefly, particles were prepared by vigorously mixing 1 mL of 20 mM AgNO_3_ with 200 μL of 0.5 mM HAuCl_4_ in 98 mL of Milli-Q (Millipore Sigma. Burlington, VT, USA) water at room temperature followed by the addition of 1 mL of 5.0 mM tannic acid with continuous mixing. The mixture turned yellow within 30 min, indicative of the formation of the AgNPs. The solution was then titrated to pH 6.0 and stored at 4 °C until use. Thiosulfate standards were made in PBS and added to the AgNPs in 96 well plates at a ratio of 30 μL PBS to 200 μL AgNPs and absorbance measured at 419 nM. All subsequent experiments were analyzed with this 30-μL PBS:200 μL AgNP ratio. Thiosulfate concentration was obtained from a standard curve of thiosulfate concentration versus (A_0_−A)/A_0_ per Dong et al. [70], where A_0_ is absorbance at 0 μM thiosulfate and A is absorbance with thiosulfate. Four wells were loaded with each standard and absorbances averaged for each concentration. Absorbance from eight experimental wells was averaged and thiosulfate concentration was determined from this value. Initial studies indicated that 60 min was sufficient for maximum changes in absorbance. Preliminary experiments showed that catalase also exhibited significant absorbance at this wavelength so a separate standard curve was made where A_0_ was the absorbance in the presence of catalase.

We previously observed that 100 and 300 μM H_2_S produced absorbance responses similar to those of 100 and 300 μM thiosulfate, indicating a direct reaction of H_2_S with AgNPs, and this effect could be substantially reduced by initially covering the wells for 120 min to allow the reaction with H_2_S to proceed and then uncovering wells for 90 min to allow the H_2_S to dissipate prior to adding the samples to AgNP. The thiosulfate that was present as a contaminant or produced by H_2_S oxidation was measured in quinone-free experiments and it was subtracted from the total thiosulfate produced by the various quinones in these experiments. We use quinones in this instance to represent all the hydroquinones, quinones and related compounds used in this study.

### 4.4. Cells

Human embryonic kidney (HEK293) cells were cultured in T-25 tissue culture flasks and maintained at 37 °C in a 5% CO_2_ humidified incubator with 21% O_2_ supplemented with DMEM (low glucose) containing 10% FBS and 1% Pen/Strep. They were transferred to 96-well plates and experiments were conducted when cells were 80–95% confluent. Fluorescence was measured on the SpectraMax M5e plate reader as described above. Compounds of interest were typically added after an initial baseline reading.

### 4.5. Oxygen Dependency of Quinone Reactions with H_2_S in Buffer

In order to determine whether polyphenols were autoxidized prior to reacting with H_2_S, we bubbled PBS with either 100% N_2_ or 100% O_2_, or used PBS equilibrated with room air (21% O_2_), and then added compounds of interest and allowed them to react for various time periods. Aliquots (200 μL) were placed in 96-well plates, SSP4 was then added and fluorescence monitored for an additional 90–130 min.

### 4.6. Chemicals

**Figure 7:**
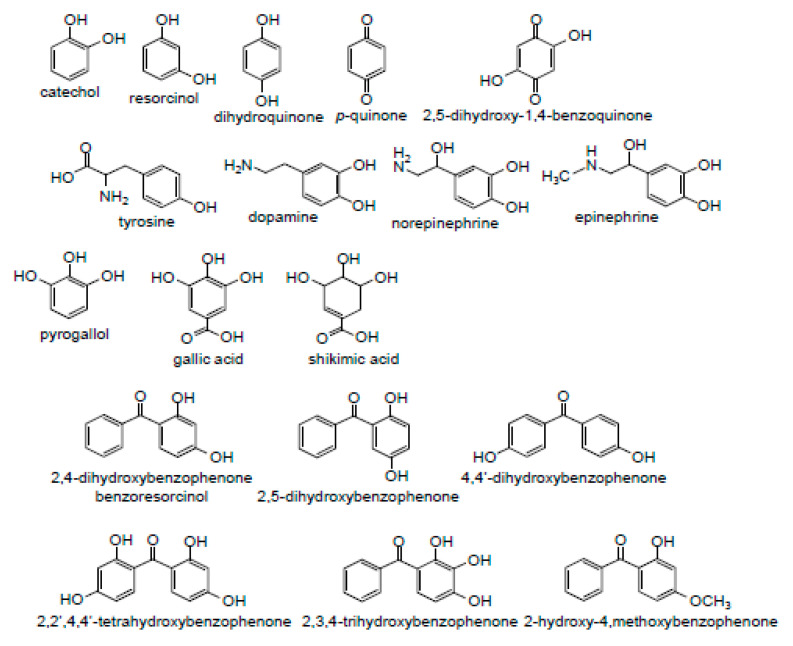
Structures of compounds used in this study.

SSP4 was purchased from Dojindo molecular Technologies Inc. (Rockville, MD, USA). All other chemicals were purchased from Sigma-Aldrich (St. Louis, MO, USA) or ThermoFisher Scientific (Grand Island, NY, USA). Compounds used in this study are shown in Figure 7. “H_2_S” is used throughout to denote the total sulfide (sum of H_2_S + HS^−^) derived from Na_2_S as S^2−^ most likely does not exist under these conditions [71]. Phosphate buffered saline (PBS; in mM): 137 NaCl, 2.7 KCl, 8 Na_2_HPO_4_, 2 NaH_2_PO_4_. pH was adjusted with 10 mM HCl or NaOH to pH 7.4.

### 4.7. Statistical Analysis

Data were analyzed and graphed using QuatroPro (Corel Corporation, Ottawa, ON, Canada) and SigmaPlot 13.0 (Systat Software, Inc., San Jose, CA, USA). Statistical significance was determined with one-way ANOVA and the Holm–Sidak test for multiple comparisons as appropriate using SigmaStat (Systat Software, San Jose, CA, USA). Results are given as mean ± SE; significance was assumed when *p* ≤ 0.05.

## Data Availability

The data presented in this study are openly available in IJMS at this paper.

## Supplementary Materials

The supplementary materials are available online at https://www.mdpi.com/1422-0067/22/2/961/s1.
