# Supplementary material for: Oxidation of Hydrogen Sulfide by Quinones: How Polyphenols Initiate Their Cytoprotective Effects
Source: Int J Mol Sci. 2021 Jan 19;22(2):961. doi: 10.3390/ijms22020961 (PMC7835830; doi:10.3390/ijms22020961)
Supplement: Supplementary file 1 [file ijms-22-00961-s001.pdf]

Supplemental Information

Oxidation of Hydrogen Sulfide by Quinones: How Polyphenols Initiate Their Cytoprotective Effects

Kenneth R. Olson[a,b], Yan Gao[a] and Karl D. Straub[c,d]

a. Indiana University School of Medicine - South Bend Center, South Bend, Indiana 46617 USA

b. Department of Biological Sciences, University of Notre Dame, Notre Dame IN 46556

c Central Arkansas Veteran's Healthcare System, Little Rock, AR 72205, USA

d Departments of Medicine and Biochemistry, University of Arkansas for Medical Sciences, Little Rock, AR 72202, USA

Running Head: Sulfur Metabolism by Quinones

Address correspondence to:

Kenneth R. Olson, Ph.D.
Indiana University School of Medicine -South Bend
Raclin Carmichael Hall
1234 Notre Dame Avenue
South Bend, IN 46617
Phone: (574) 631-7560
Fax: (574) 631-7821
e-mail: kolson@nd.edu

Supplemental Figures

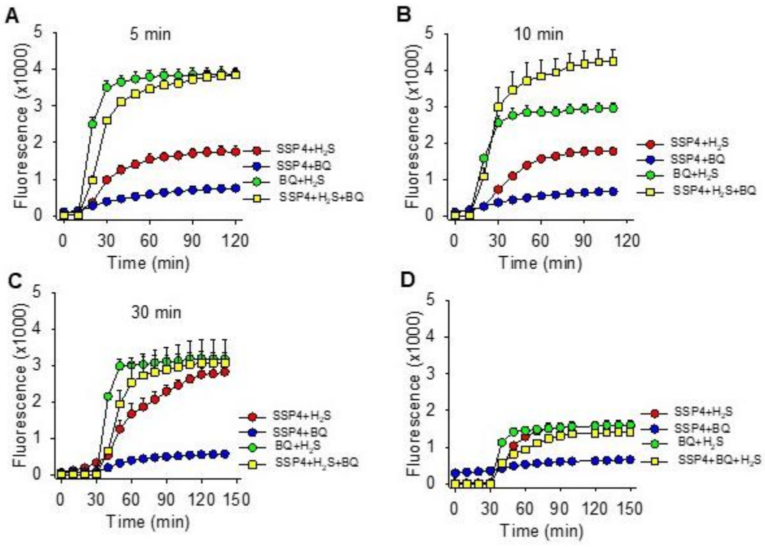

Supplemental Figure S1. Polysulfide formation (SSP4 fluorescence) after 5 (**A**), 10 (**B**) or 30 (**C**) min delayed addition of one of the three substrates in the reaction of 5 μM SSP4, 100 μM *p*-benzoquinone (BQ) and 300 μM H_2S compared to rapid sequential addition of SSP4, H_2S and BQ (SSP4+H_2S+BQ). Initial two substrates are shown in figure. The rate of polysulfide formation was greatest when BQ and H_2S were added prior to SSP4. (**D**) Adding BQ to SSP4 prior to H_2S nearly completely prevented the increase in SSP4 fluorescence. Sequential addition of SSP4, H_2S and BQ was sufficient to overcome the inhibitory effect of BQ. Mean +SE, $n = 4$ wells per treatment.

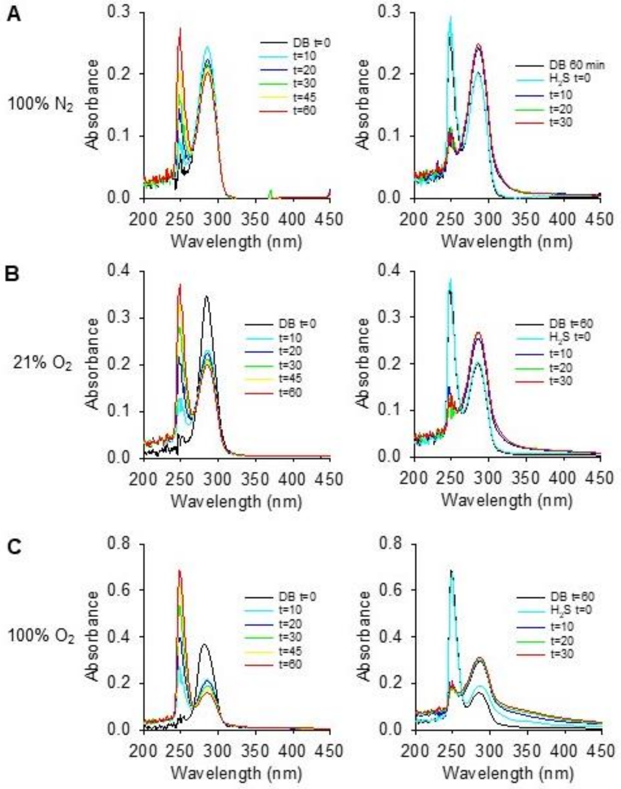

Supplemental Figure S2. Effects of O_2 alone (left panels) and in combination with 100 μM H_2S (right panels) on absorption spectra of 100 μM 1,4-dihydroxybenzamine (DB). Buffer was bubbled with 100% N_2 (**A**), 21% O_2 (**B**) or 100% O_2 (**C**) for 20 min prior to addition of compounds. In 100% N_2 there is only a slight decrease in absorption at 289 nm indicatong very little oxidation of DB; a slight amount of BQ (249 peak) is also observed. Addition of 100 μM H_2S reduces nearly all of this BQ to DB. As the percent O_2 increases there is a progressive increase in DB oxidation shown by the decrease in the 289 nm peak and increase in the 249 peak (note increase in absorbance axis with increasing O_2). BQ was reduced to DB after addition of H_2S.

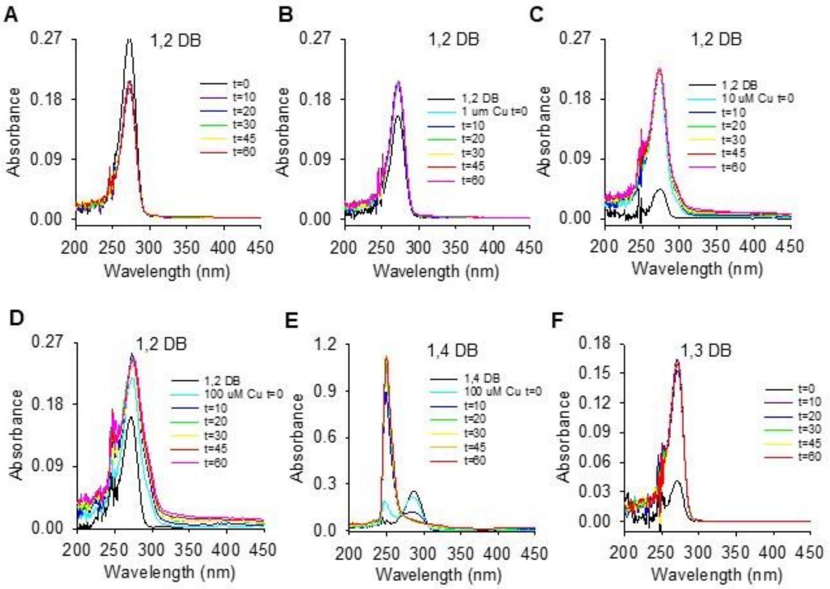

Supplemental Figure S3. Spectral analysis of autoxidation of dihydroxybenzenes. 1,2-dihydroxybenzene (1,2DB) is not autoxidized in 21% O_2 (**A**) nor in 21% O_2 in the presence of 1, 10 or 100 μM $CuCl_2$ (**B**-**D**), whereas 1,4-DB is readily oxidized by 100 μM $CuCl_2$ (**E**). (**F**) 1,3-DB is not autoxidized in 21% O_2.

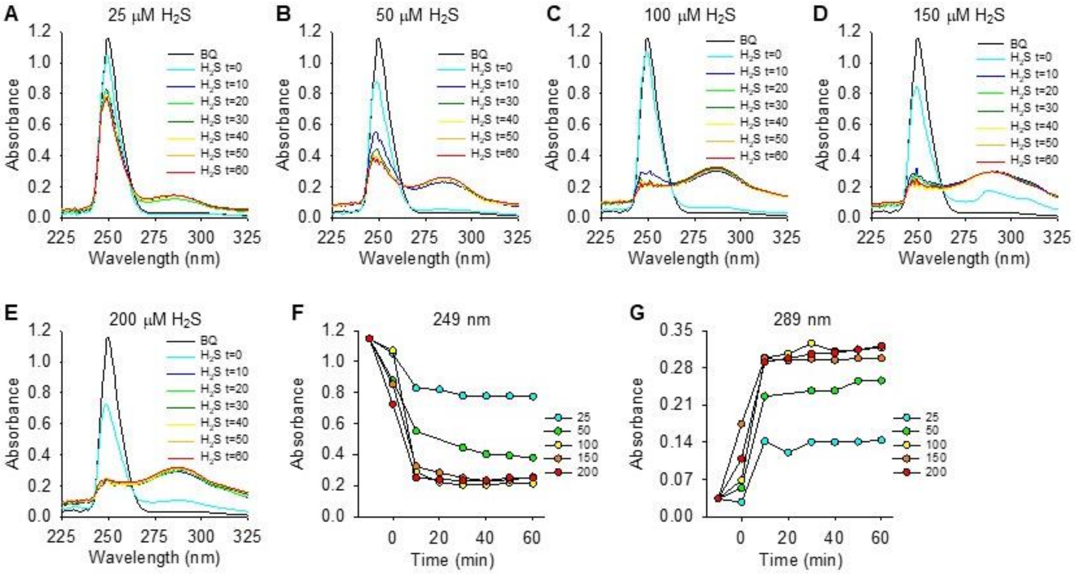

Supplemental Figure S4. Effects of increasing H_2S concentration (25-200 μM, **A-E**, respectively) on the absorption spectra of 100 μM 1,4-benzoquinone (BQ). The BQ absorbance peak at 249 nm is concentration-dependently eliminated and replaced by a peak at 289 nm consistent with formation of 1,4-dihydrobenzene when H_2S >100 μM. (**F-G**) show time course of absorption spectra at 249 and 289 nm, respectively at different H_2S concentrations; BQ spectrum prior to H_2S is shown at -15 min. The reaction is essentially complete by 10 min when H_2S >50μM.

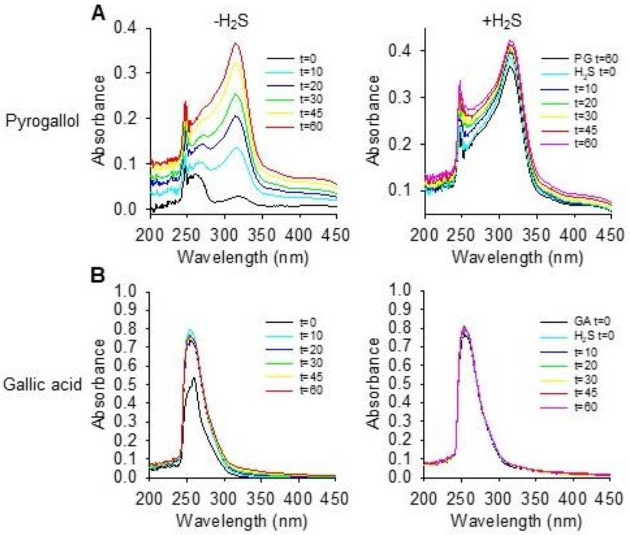

Supplemental Figure S5. Absorption spectra of 100 μM pyrogallol (PG; **A**) and 100 μM gallic acid (GA; **B**) in 21% O_2 for 60 min before (left panels) and 60 min after addition of 300 μM H_2S (right panels). An initial PG peak at ~560 nm decreases in 21% O_2 and is replaced by a strong absorbance peak at 320 nM that is not affected by subsequent addition of H_2S. The absorbance peak of GA at 260 nm appears to be unaffected by exposure to either O_2 or H_2S.

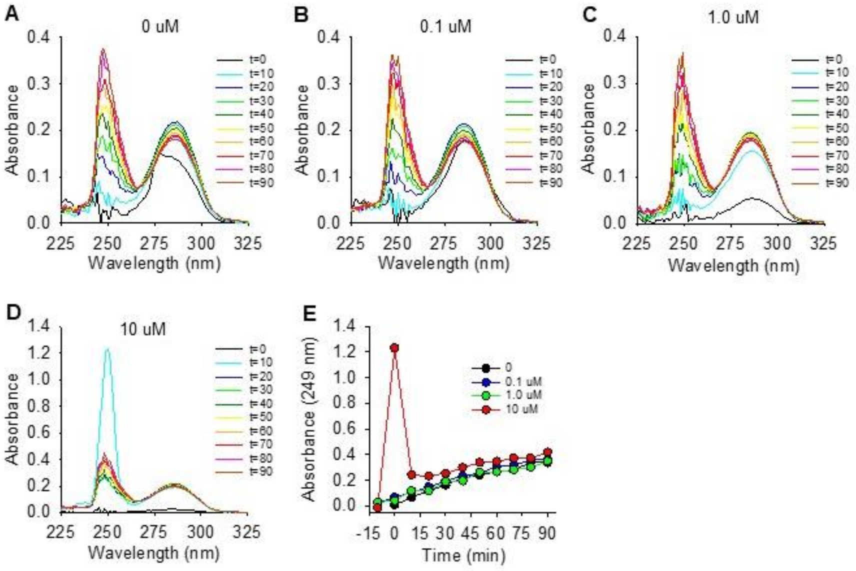

Supplemental Figure S6. (**A-D**) Absorption spectra of 100 μM 1,4-dihydroxybenzamine (DB) and variable concentrations of 1,4-benzoquinone (BQ). (**E**) Time-dependent absorption at 249 nm as a function of BQ concentration.

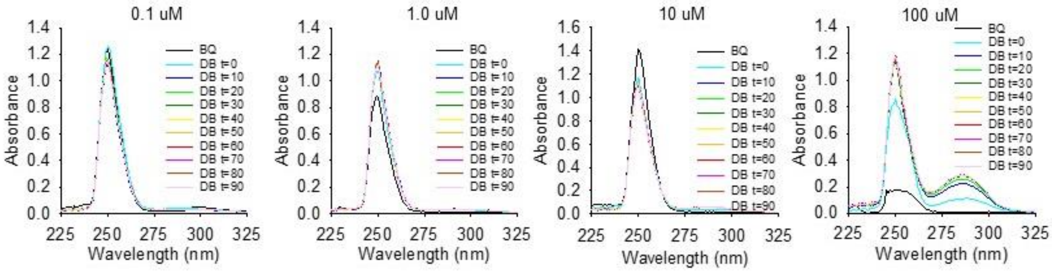

Supplemental Figure S7. Absorption spectra of 100 μM 1,4-benzoquinone (BQ) and variable concentrations of 1,4-dihydroxybenzamine (DB).

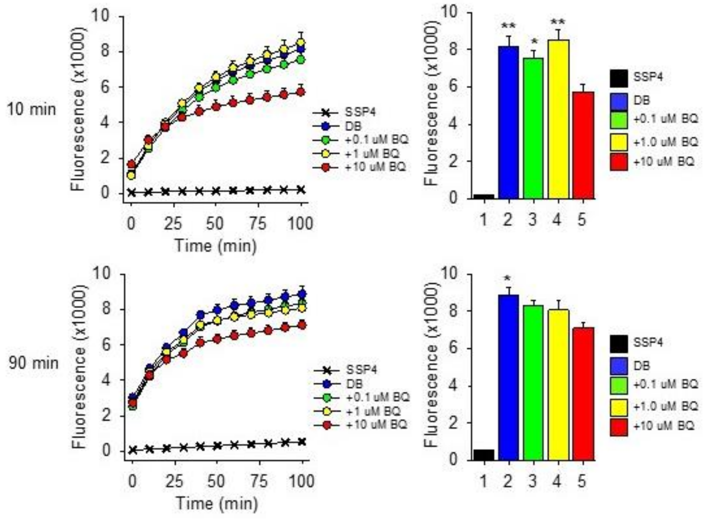

Supplemental Figure S8. Effects of variable 1,4-benzoquinone (BQ) concentration on polysulfide production (SSP4 fluorescence) by 100 μM 1,4 dihydroxybenzene (DB) and 300 μM H_2S. DB and BQ were added and allowed to react for 10 min (**A**) or 90 min (**B**) prior to addition of H_2S; SSP4 was added 10 min after H_2S. Neither 0.1 nor 1.0 μM BQ affected polysulfide production compared to 0 μM BQ, whereas 10 μM BQ inhibited it irrespective of DB-BQ incubation period. Mean +SE n=4 wells per treatment; *, *p<0.05*; **, *p<0.01*, significantly different from 10 μM BQ.

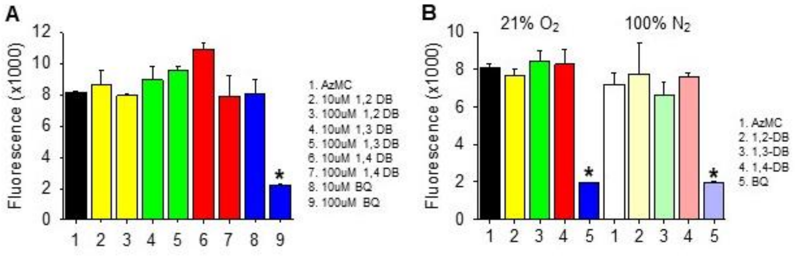

Supplemental Figure S9. Neither dihydroxybenzenes (DB) nor *p*-benzoquinone (BQ) produce H_2S (AzMC fluorescence) from 300 μM mixed polysulfide (K_2S_n) in 21% O_2 or 100% N_2. (**A**)

Effects of 10 and 100 μM DB and BQ at 21% O_2. (**B**) comparison of 100 μM DB and BQ at 21% O_2 and 100 % N_2. BQ at 100 μM significantly (*p<0.001*) inhibited AzMC fluorescence in all instances suggesting BQ inhibits the reaction between H_2S (present in K_2S_n) and the fluorophore. Bar graphs show fluorescence at 130 min. Mean +SE, n = 4 wells per treatment; *, *p<0.001* compared to AzMC with K_2S_n (AzMC).

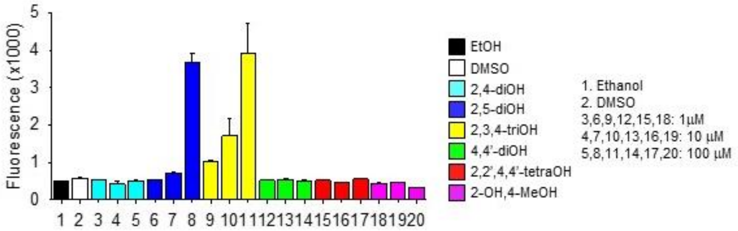

Supplemental Figure S10. Polysulfide production (SSP4 fluorescence) after 120 min incubation from 300 μM H_2S by di-, tri, and tetra-hydroxyphenones and 2-hydroxy,4methoxyphenone. Only 2,5-dihydroxyphenone and 2,3,4-trihydroxyphenone oxidized H_2S to polysulfides. Phenone concentration (1, 10, 100 μM) increases from left to right; mean +SE; *n* = 4.
